# Development of a Dissemination Platform for Spatiotemporal and Phylogenetic Analysis of Avian Infectious Bronchitis Virus

**DOI:** 10.3389/fvets.2021.624233

**Published:** 2021-05-04

**Authors:** Manuel Jara, Rocio Crespo, David L. Roberts, Ashlyn Chapman, Alejandro Banda, Gustavo Machado

**Affiliations:** ^1^Department of Population Health and Pathobiology, College of Veterinary Medicine, North Carolina State University, Raleigh, NC, United States; ^2^Department of Computer Science North Carolina State University, Raleigh, NC, United States; ^3^Poultry Research and Diagnostic Laboratory, College of Veterinary Medicine, Mississippi State University, Pearl, MS, United States

**Keywords:** infectious bronchitis, virus evolution, outbreak analytics, avian disease, evolutionary epidemiology

## Abstract

Infecting large portions of the global poultry populations, the avian infectious bronchitis virus (IBV) remains a major economic burden in North America. With more than 30 serotypes globally distributed, Arkansas, Connecticut, Delaware, Georgia, and Massachusetts are among the most predominant serotypes in the United States. Even though vaccination is widely used, the high mutation rate exhibited by IBV is continuously triggering the emergence of new viral strains and hindering control and prevention measures. For that reason, targeted strategies based on constantly updated information on the IBV circulation are necessary. Here, we sampled IBV-infected farms from one US state and collected and analyzed 65 genetic sequences coming from three different lineages along with the immunization information of each sampled farm. Phylodynamic analyses showed that IBV dispersal velocity was 12.3 km/year. The majority of IBV infections appeared to have derived from the introduction of the Arkansas DPI serotype, and the Arkansas DPI and Georgia 13 were the predominant serotypes. When analyzed against IBV sequences collected across the United States and deposited in the GenBank database, the most likely viral origin of our sequences was from the states of Alabama, Georgia, and Delaware. Information about vaccination showed that the MILDVAC-MASS+ARK vaccine was applied on 26% of the farms. Using a publicly accessible open-source tool for real-time interactive tracking of pathogen spread and evolution, we analyzed the spatiotemporal spread of IBV and developed an online reporting dashboard. Overall, our work demonstrates how the combination of genetic and spatial information could be used to track the spread and evolution of poultry diseases, providing timely information to the industry. Our results could allow producers and veterinarians to monitor in near-real time the current IBV strain circulating, making it more informative, for example, in vaccination-related decisions.

## Introduction

Infectious bronchitis is caused by the worldwide distributed avian gammacoronavirus infectious bronchitis virus (IBV) (1), a highly contagious disease that a?ects the respiratory, renal, and reproductive system of poultry (2). IBV is one of the most acute and highly contagious, most prevalent, and widely disseminated diseases affecting domestic birds (3). IBV is broadly distributed within commercial poultry operations (3), and its global economic impact has been estimated to be second after the highly pathogenic avian influenza (4). This virus circulates in the form of many, ever-changing variants, some of which are globally distributed, while others are found only in specific locations and countries (5). Vaccination for IBV has been shown to produce limited cross-protection among serotypes (6, 7); therefore, it is pivotal to timely monitor IBV strain circulations, while effective vaccination programs are the main control strategy the poultry industry utilizes. The information generated from diagnostic laboratories has been historically used to monitor disease spread on livestock (8, 9), mostly in swine (10–13), cattle (14–16), sheep (17), and goats (18). Likewise, several studies have been focused on studying the extent and prevalence of infectious diseases affecting wild birds, such as influenza A viruses (19–22), West Nile and Usutu virus (23, 24), and Newcastle disease virus (25). However, it has not been utilized to track non-reportable diseases in poultry populations, such as IBV infections. Therefore, sequence data generated from the diagnosis of diseases in poultry populations such as IBV could be used to track its spatial spread and evolution, contributing to the development of more timely and integrative control strategies against IBV. Knowledge about IBV circulation among flocks could help in outbreak investigations and provide relevant information for vaccination strategies (26–28). However, due to the continuous changes in poultry production, improvements in vaccine technologies, or the emergence of novel and recombinant field viruses among others, this information is never static and keeps changing regularly. Therefore, to attain meaningful interpretations and actionable results, there is a need to monitor the dynamics of viral spread. Recently, epidemiological models have been coupled with phylogenetic methods to track the spatial and temporal spread of infectious agents, promoting the development of phylodynamics (29–32). The importance of phylogeography and phylodynamics on characterizing the evolutionary history of IBV has been previously described, pointing out the direct impact of IBV control and poultry production (33–35). These fields have seen the development of analytical methods that now can take advantage of genetic data to characterize the spatiotemporal and evolutionary patterns of infectious diseases by reconstructing how pathogens spread across landscapes (36–39).

The development of modern data visualization platforms such as Nextstrain (40) allows the simultaneous analysis of phylogenetic, epidemiological, and geographic information (41). These tools have gained special importance, allowing us to understand the spread of viral diseases, such as Avian flu H7 (42), and most recently, the pandemic SARS-CoV-2 (43–45).

In this study, we explore the local dispersal of IBV within the poultry farms. We described the spatiotemporal spread and evolution of 132 IBV samples across one state of the United States and additionally built an interactive web visualization dashboard based on the Nextstrain platform. Our derivative of the Nextstrain platform provides a portal for complete phylodynamic analysis by research staff while affording producers' redacted views into the origins, spread, and phylodynamic analysis that preserve anonymity across producers who contribute samples for analysis but enable producers to learn valuable information about their operations.

## Methods

### Data

We gathered IBV isolates from 2016 to 2019 from 52 poultry farms in North Carolina. Oropharyngeal swab samples of sick birds were tested at the Mississippi Poultry Research and Diagnostic Laboratory (PRDL) (79 samples) or the North Carolina Veterinary Diagnostic Laboratory System (NCVDLS) (53 samples). In all cases, IBV presence was confirmed by real-time reverse transcriptase PCR (RTPCR) (46). From the positive samples, RNA extraction was performed, and the hypervariable region of the S1 gene was amplified and used for phylogenetic studies. For the PRDL samples, the viral RNA extraction and RT-PCR were conducted using the 5X MagMaxTM Pathogen RNA/DNA extraction kit of Applied Biosystems, as previously described (47). The primers used for the S1 gene amplification were NEWS1OLIGO5′ and S1OLIGO3′ that amplify a product ranging between positions 1,730 and 1,750 sequences and between positions 20,300 and 22,041—depending of the sequence (47). For the NCVDLS samples, the RNA was extracted using the Invitrogen TRIzol™ reagent according to the manufacturer guidelines (ThermoFisher Scientific, NY). The RT-PCR was conducted with the High-Capacity RNA-to-cDNA™ kit (ThermoFisher Scientific). The sequences for the RT-PCR were forward primer 5′TGCTTCCTTTATAGGCATCGGT3′ and reverse primer 5′GGAATGATGCCAAAGCACCC3′ that amplifies a 1,099-base sequence between positions 22,778 and 23,779, depending on the sequence. The cycling conditions for both primer sets were as previously described by Jackwood et al. (47). The only exception was that for the primers used for the NCVDLS samples, the annealing temperature was set at 55°C, and the elongation temperature was set at 72°C. The S1 amplicons were gel purified on a 1% agarose gel and sent to GenWiz for sequencing. Additionally, we collected the following information: farm location, serotype to which each sequence belongs, and all applied vaccines (Supplementary Table 1).

Sequences were aligned using Mega X, available at www.megasoftware.net (48). The recombination detection program (RDP) v4 was used to search for evidence of recombination within our dataset (49). The alignment was screened using six different methods (BootScan, Chimaera, MaxChi, RDP, 3Seq, and SiScan). We found four recombinant samples, which were removed from the downstream analysis, from those sequences: two were identified as serotype Georgia 98, and the remaining ones as Georgia 13 and Arkansas DPI. To determine whether there was a sufficient temporal molecular evolutionary signal of the IBV sequences, we used TempEst v1.5 (50). To calculate the *P*-values associated with the phylogenetic signal analysis, we used the approach described by Murray et al. (51) based on 1,000 random permutations of the sequence sampling dates (52). The relationship found between root-to-tip divergence and sampling dates (years) supported the use of molecular clock analysis in this study. Root-to-tip regression results supported a significant temporal signal (*R*^2^ = 0.43, *p* < 0.05).

To assess how our sequences relate to previously published sequences from other parts of the country, we used 35 IBV S1 gene sequence data from the United States collected from GenBank (53), available at https://www.ncbi.nlm.nih.gov/nucleotide/ (accession numbers found in (Supplementary Table 2).

### Phylogeographic Analysis

Phylogenetic trees were estimated by Bayesian inference through Markov chain Monte Carlo (MCMC), implemented in BEAST v2.5.0 (54). By using ModelFinder (55) built into IQ-Tree version 1.6.1 (56), we compared different substitution models to find the one that showed the best fit for our genetic dataset. The marginal likelihood value supported the use of the general time-reversible model (HKY) with gamma-distributed rate heterogeneity plus a proportion of invariable sites (HKY + F + G4) (57) (Supplementary Table 3). Phylogeographic history of IBV dispersal was recovered from the obtained spatiotemporal phylogeny. Phylogenetic trees were generated by a discrete phylogeography estimation by Bayesian inference through Markov chain Monte Carlo (MCMC), implemented in BEAST v2.5.0 (54). Finally, we applied a separate Hasegawa–Kishino–Yano (HKY+G) (58) substitution model with gamma-distributed rate heterogeneity among sites (59).

### Phylodynamic Analysis

Following the analytical framework described by Dellicour et al. (60) and using the R package “seraphim” (61), we extracted the spatiotemporal information from 100 input trees sampled at regular intervals from the post-burn-in posterior distribution to account for phylogenetic uncertainty (62, 63). Each phylogenetic branch was then summarized and represented as a distinct vector defined by its start and end location and dates (60, 61), converting each of these branches in a representation of an independent viral lineage dispersal event (62, 64).

**Figure 1:**
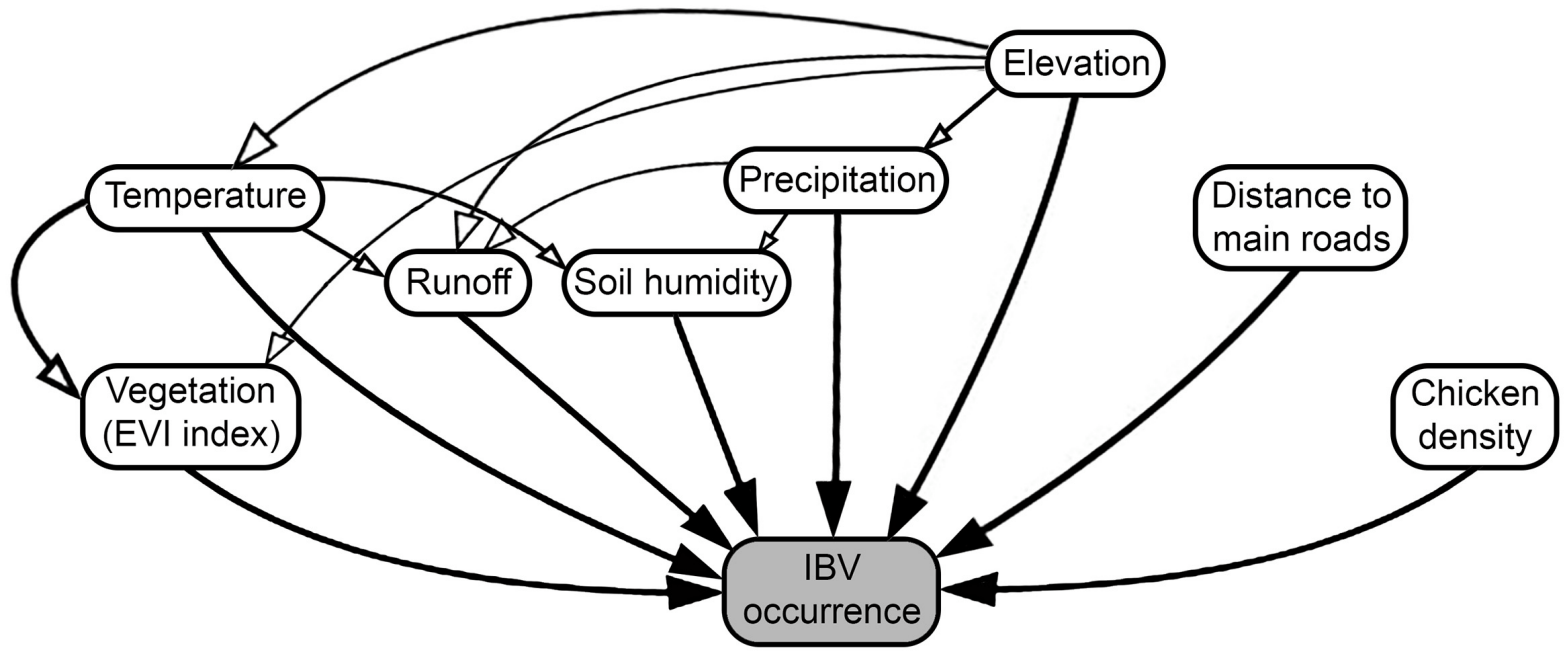
Diagram of factors promoting or restricting infectious bronchitis virus (IBV) between-farm dispersion. Arrows represent the relationship between the predictor and IBV occurrences, where white arrows represent indirect, and black arrows direct relationships.

To determine the effect of different predictors in the spread of IBV, we collected the outputs generated by the previous continuous phylogeographic analysis, then used the analytical framework developed by Dellicour et al. (60), considering a continuous location approach (based on the geographic coordinate location of each poultry farm) and a random walk diffusion model. Spatiotemporal information was extracted from 100 trees sampled at regular intervals from the posterior distribution (after burn-in) to account for phylogenetic uncertainty. Each phylogenetic branch was considered a vector defined by its start and end location (latitude and longitude), and its start and end dates. Thus, each phylogenetic branch represents a conditionally independent viral lineage dispersal event (64). Statistical significance for the correlation between phylogenetic and predictive factors was tested using 100 trees generated and expressed in the form of Bayes factors (BF) (60). We approximated a BF value for each combination of predictors tested. For the interpretation of BF values, predictive factors were treated either as a conductance (variables that promote the spread of the disease) or a resistance factor (variables that impede its spread), then we used (65) to determine the level of impact of each variable on the spread of IBV. Therefore, BF values between 3 and 20 were considered “positive,” values between 201 and 50 were considered “strong,” and values >150 were considered “very strong” (65). This analysis was performed using the R package “seraphim” version 1.0 (61). Thus, we included the environmental condition of each farm location here represented by the annual temperature, annual precipitation, elevation, runoff (an index of the quantity of water discharged in surface streams), wetness index (commonly used to quantify topographic control on hydrological processes) (66), enhanced vegetation index (EVI) (widely used to quantify vegetation greenness), chicken density, and distance to the main roads (Figure 1 and Supplementary Table 4 for more details). Briefly, the distance between each farm (geographic coordinates) to the nearest main roads was calculated based on the Euclidean distances.

### Near Real-Time Visualization

**Figure 2:**
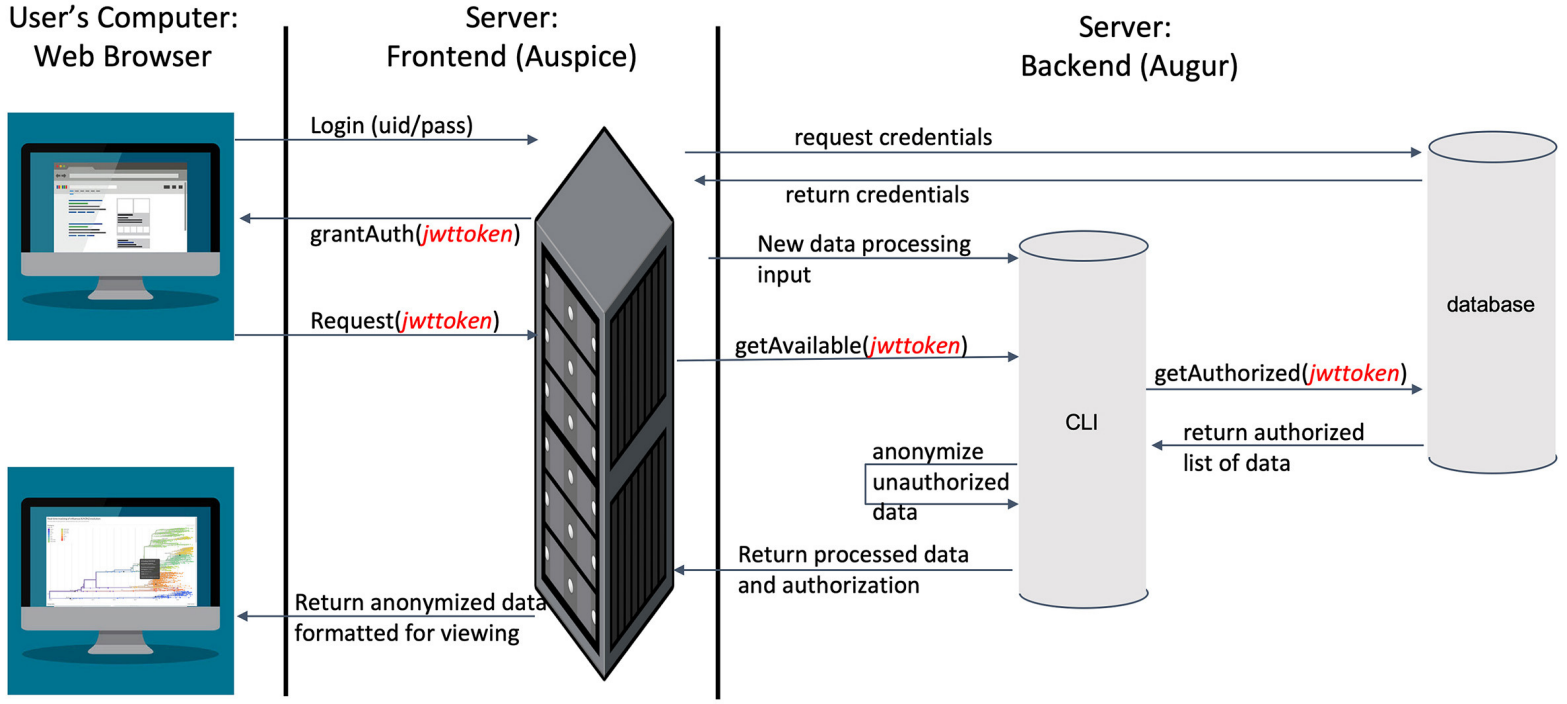
An overview of the modified Nextstrain architecture, leveraging industry-standard SSL encryption, JSON web tokens for identity management, and separate servers for web hosting and data storage to ensure information security.

To visualize the evolution and spread of IBV in an interactive tool that allows for near real-time updates using phylogenetic, spatial, and epidemic information based on outbreak data (Supplementary Table 5), we used Nextstrain (https://nextstrain.org) (40). IBV sequences were processed in Nextstrain's platform using the Augur bioinformatics toolkit (augur) (67) and visualized using Auspice (https://github.com/nextstrain/auspice) (68). Auspice is designed to provide a detailed view of the results of the Augur analysis. This level of detail is appropriate for researchers studying population and geographic trends but may provide more information than producers contributing viral samples are willing to share more broadly. To balance between the needs of detailed analyses and a desire to preserve producers' anonymity, we modified Auspice to automatically redact certain details based on users' access rights we defined (Figure 2).

**Figure 3:**
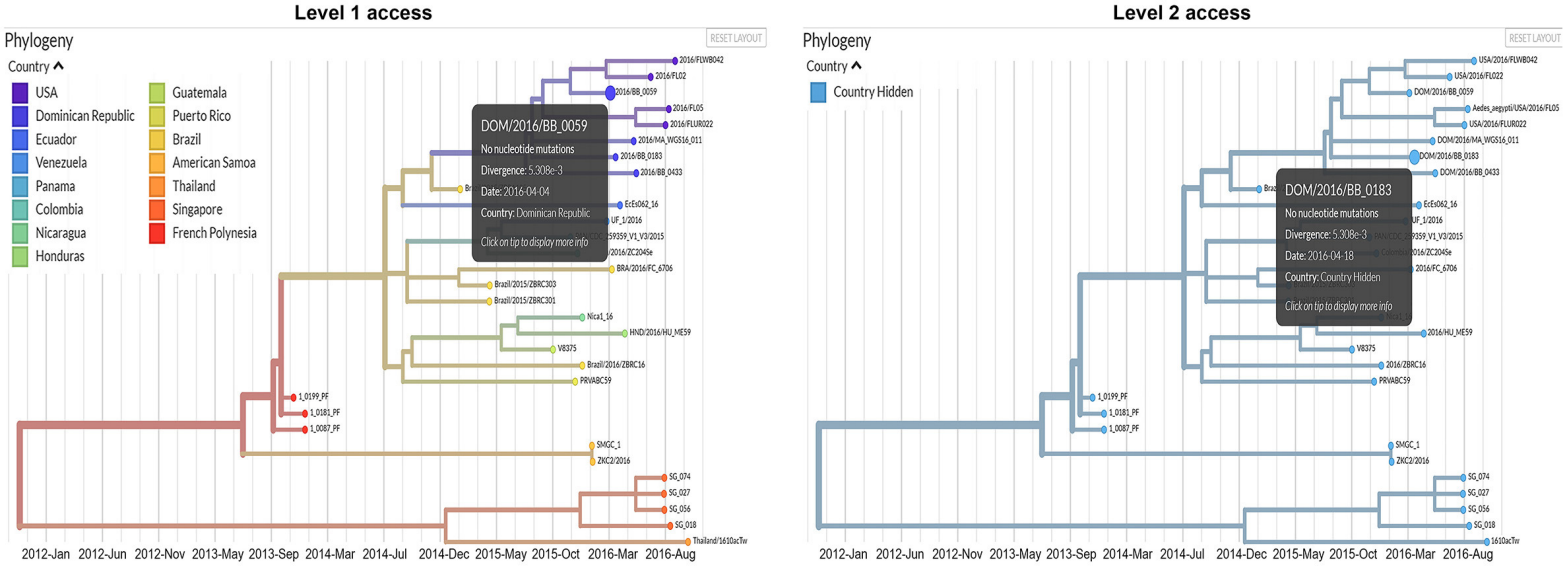
Comparison between the information that will be available for users with level 1 and level 2 access permissions.

In the modified dashboard (Figure 2), each dataset has three levels of access. A user has (1) unrestricted access to the data visualization, (2) complete data visualization without location data, and/or (3) no access to the data visualization. After a user registers a new account with the system, the project staff grants full access to the data they provide (level 1), and redacted access to data provided by other producers (level 2) (Figure 3) is an overview of the process by which users' access is verified and data anonymity is ensured. When a user logs in, a set of authorization credentials are retrieved from a database located on a separate server, and a list of available data sets are returned for display in the user's browser. In addition, a JSON Web Token (JWT) (69) is created for the user, which cryptographically encodes their access in a secure data structure and allows the system to verify access.

The user is then able to request the visualization for any data set they have access to by clicking the link in their browser. The resulting REST API request is made via the Auspice web server, which is then routed to the Auspice CLI backend where the JWT is used as a unique identifier in the database to determine the level of access. For datasets that users have level 2 access to, the backend server redacts all location and sensitive information prior to returning the response to the frontend web server—no sensitive information is allowed to leave the backend server if a user is unauthorized to view it. The visualization information is then returned from the frontend web server to the client where their browser renders it for viewing. Figure 3 below illustrates the difference between levels 1 and 2 access visualizations.

This architecture is designed specifically to enhance data security and preserve anonymity. First, by leveraging industry-standard JWT technology to cryptographically sign identity verification, we can ensure that individuals requesting data are rightfully accessing it. Second, by separating the frontend and backend servers, we are able to leverage strict isolation between the data (which resides on the backend server) and any publically available services (which reside on the frontend server). Strict firewall and access policies ensure the backend server has a far lower attack surface. Furthermore, this separation ensures that when data leaves the backend server, they are already anonymized before going out over the Internet, providing an additional layer of security beyond that provided by SSL encryption for all web traffic.

## Results

**Figure 4:**
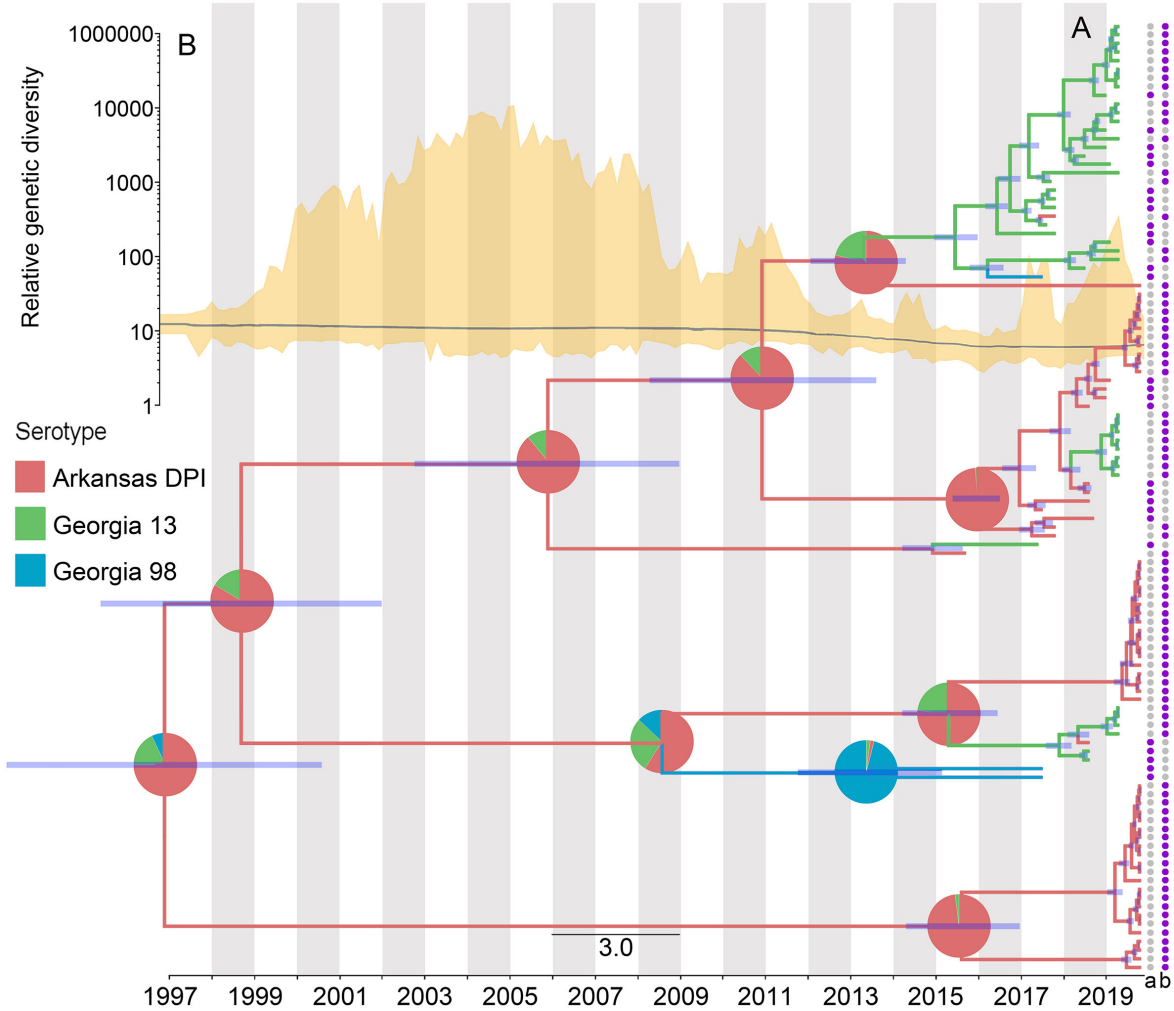
Dispersal history of IBV lineages, as inferred by discrete phylogeographic analysis. **(A)** Maximum clade credibility phylogeny colored according to the IBV strain to which each sequence belongs. Branch bars represent posterior probabilities of branching events (*P* > 0.95). The probabilities of ancestral states (inferred from the Bayesian discrete trait analysis) are shown in pie charts on each node, representing the most likely IBV strain. Purple points at the end of the phylogenetic tips represent the applied vaccine on the farm where the sequences were collected, where a = MILDVAC-MASS+ARK, and b = Data not available. **(B)** Spatiotemporal patterns in the relative genetic diversity represented through the Bayesian SkyGrid plot (yellow); the mean estimate is represented by the dark line, while the shaded light yellow regions correspond to the 95% highest posterior density (HPD), and in the left, the y-axis shows the number of sampled sequences over time.

The results of the phylogeographic analyses estimated the time of the most recent common ancestor for the studied IBV sequences as between 2005 and 2006. The ancestral reconstruction showed that the most likely strain of origin in North Carolina poultry farms was Arkansas DPI, with a root state posterior probability (RSPP) = 0.68, followed by Georgia 13 (RSPP = 0.24), and Georgia 98 (RSPP = 0.09) (Figure 4A). Phylodynamic patterns of IBV spread, visualized through SkyGrid plot, showed a constant decrease in the relative genetic diversity that has been maintained until March 2019 (Figure 4B). Spatiotemporal analysis of the analyzed sequences showed an estimated median value for the mean branch velocity of 12.3 km/year (95% highest posterior density HPD = 2.7–31.1).

The vaccination program carried out on the farms was not specified for most of the collected sequences (74.5%). However, from the ones with available information (25.5%), MILDVAC-MASS + ARK was the vaccine that was applied on all farms (Figure 4A).

### Phylogeographic Diffusion in a National Context

**Figure 5:**
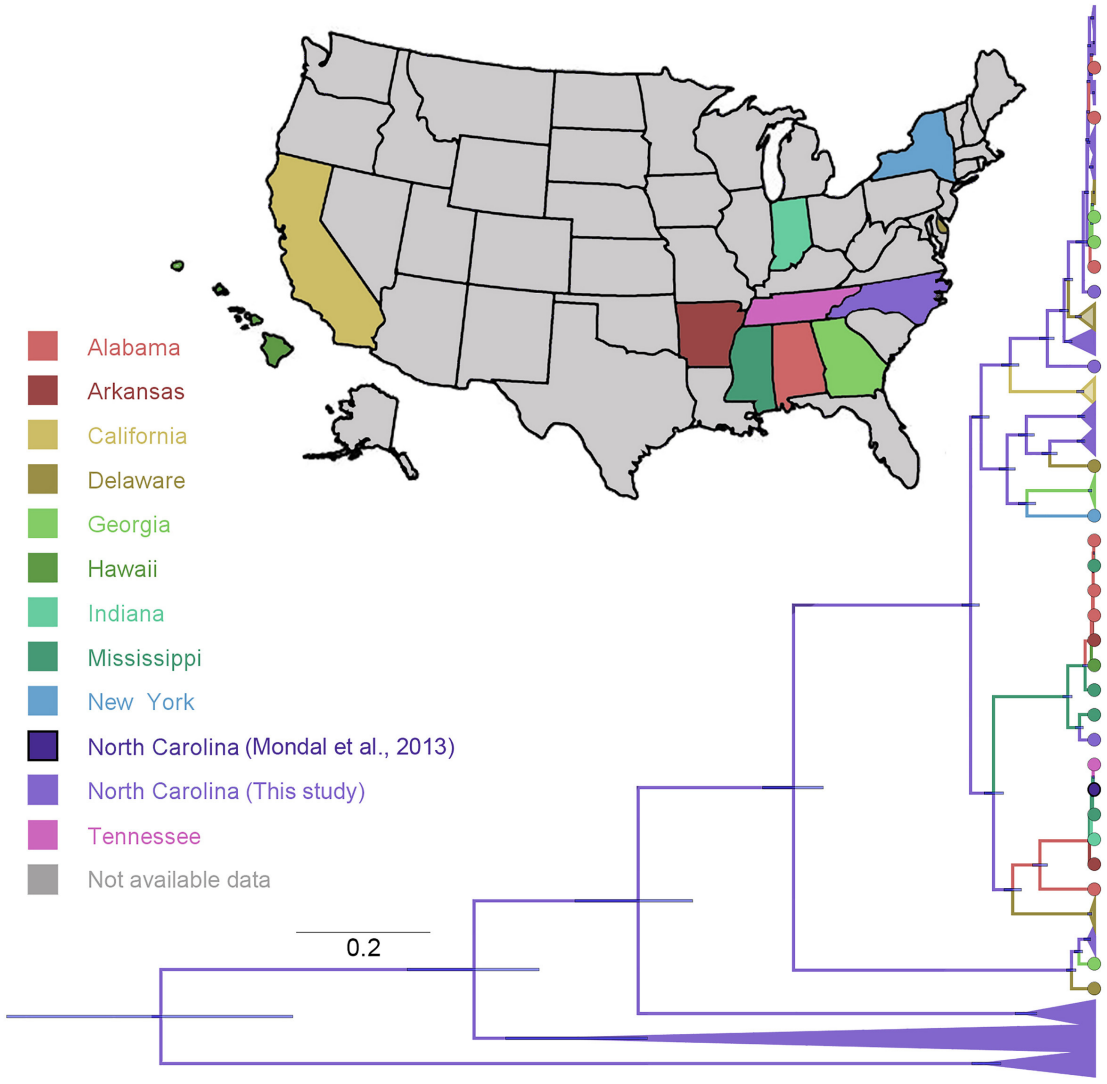
Phylogenetic reconstruction of IBV comparing the data generated in this study with data available of IBV sequences collected in the United States and available in GenBank (53). The maximum clade credibility phylogeny is colored according to the states of origin. Branch bars represent posterior probabilities of branching events (*P* > 0.95).

In addition, we confronted our samples with publicly available S1 gene IBV information nationwide (Figure 5). We found that 67% of our sequences appear widely distributed through the IBV phylogeny, while 43% is divided into three clusters showing a close relationship with sequences coming from Alabama, Georgia, and Delaware IBV sequences. On the other hand, sequences from California were the most genetically distant from the ones generated in this study. Interestingly, sequences previously collected in North Carolina (70) were more similar to sequences from other eastern states than with the sequences analyzed in this study (Figure 5).

### Factors Promoting or Restricting Infectious Bronchitis Virus Dissemination

To quantify the impact of the proposed factor on IBV distribution (Figure 1), we calculated the association between lineage duration and each covariate scaled distance through the *Q* statistic method. Our results showed that two of the variables analyzed, precipitation and runoff, had positive Q values when they were treated as resistance factors. However, they did not show enough predictive Bayes factor support; therefore, these variables should not be considered as good predictors for IBV dissemination (Table 1).

**Table 1:** List of factors promoting or restricting predictors of infectious bronchitis virus (IBV) dissemination.

**Factor**	**Regression coefficient**	***Q* statistic**	***p*(Q > 0)**	**BF**
Temperature (C)	0.046 (0.001, 0.134)	0.000 (−0.002, 0.002)	0.49	–
Temperature (R)	0.045 (0.001, 0.168)	0.000 (−0.001, 0.002)	0.72	–
Precipitation (C)	0.044 (0.001, 0.127)	−0.002 (−0.006, 0.001)	0.11	–
**Precipitation (R)**	**0.048 (0.002, 0.148)**	**0.002 (−0.001, 0.008)**	**0.92**	**1.08**
Elevation (C)	0.048 (0.002, 0.201)	0.007 (−0.036, 0.085)	0.72	–
Elevation (R)	0.030 (0.000, 0.165)	−0.004(−0.067, 0.044)	0.3	–
Vegetation (C)	0.044 (0.002, 0.144)	−0.001 (−0.008, 0.004)	0.34	–
Vegetation (R)	0.044 (0.000, 0.154)	0.001 (−0.005, 0.007)	0.77	–
Soil humidity (C)	0.051 (0.002, 0.217)	0.004 (−0.003, 0.021)	0.87	–
Soil humidity (R)	0.040 (0.000, 0.164)	−0.002 (−0.015, 0.007)	0.29	–
Runoff (C)	0.031 (0.000, 0.139)	−0.009 (−0.030, 0.000)	0.05	–
**Runoff (R)**	**0.052 (0.001, 0.164)**	**0.006 (−0.005, 0.018)**	**0.93**	**1.73**
Chicken density (C)	0.053 (0.001, 0.303)	0.011 (−0.064, 0.224)	0.36	–
Chicken density (R)	0.053 (0.000, 0.298)	0.010 (−0.061, 0.221)	0.59	–
Distance to main roads (C)	0.041 (0.000, 0.093)	−0.001 (−0.008, 0.001)	0.82	–
Distance to main roads (R)	0.019 (0.000, 0.008)	−0.009 (−0.066, 0.001)	0.19	–
Wild birds (C)	0.069 (0.038, 0.139)	0.007 (−0.053, 0.061)	0.78	–
Wild birds (R)	0.022 (0.009, 0.055)	−0.000 (−0.002, 0.001)	0.26	–

*All variables were evaluated as potential conductance (C) factors if they facilitate the dissemination of IBV, or resistance (R) if they impede its dissemination. The Bayes factor (BF) supports were only reported when p(Q > 0) is at least 90%. All the variables that reached that level appear in bold. BF results represent the level of significance, where BF > 20 is strong support, BF = 3–19 is positive support, and BF <3 is negligible*.

### Near Real-Time Visualization of Infectious Bronchitis Virus Spread Using the Nextstrain Platform

**Figure 6:**
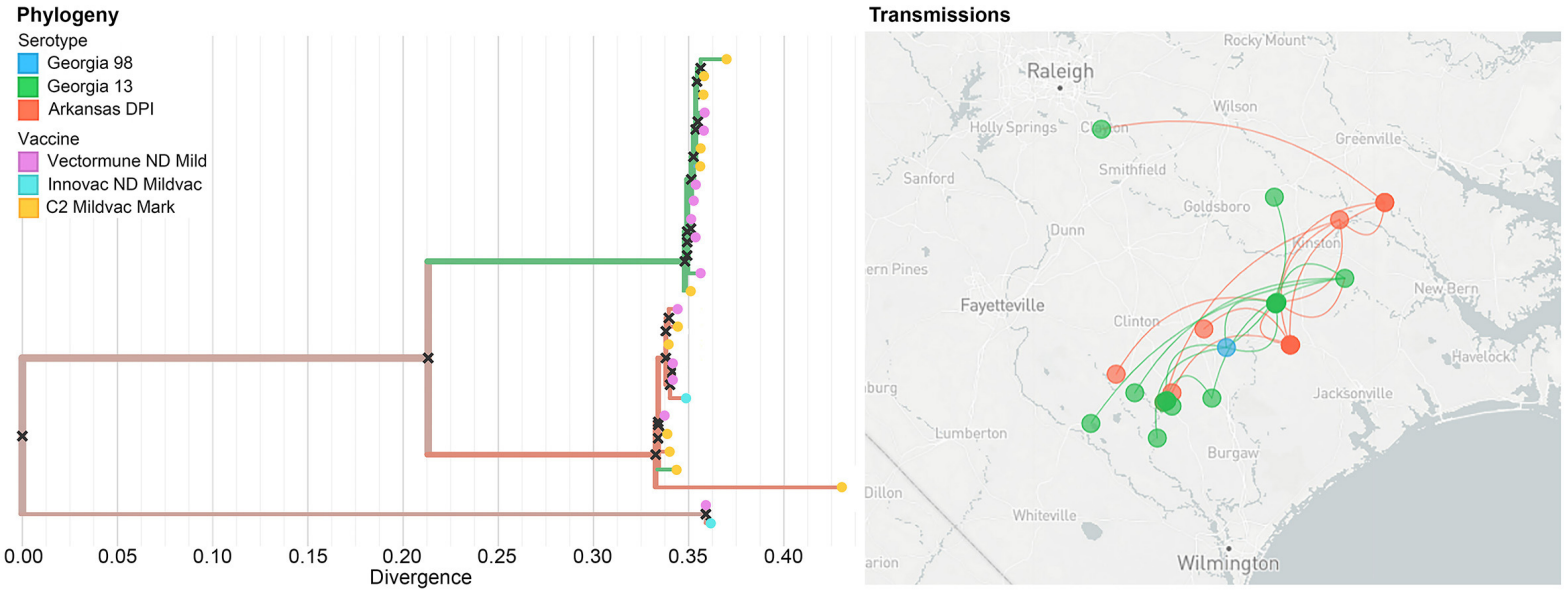
Example of the unrestricted access to the data visualization (level 1) showing the phylogeographic spread of IBV within the study area. The colors of the branches on the phylogenetic tree (left panel), as well as in the points on the map (right panel) represent different strains. In the left panel, dots at the end of the phylogenetic tree represent the vaccine that has been applied in the farms where the IBV has been collected.

Nextstrain is a publicly accessible open-source tool that can be used for near real-time interactive tracking of pathogen spread and evolution (40). We utilized the Nextstrain platform to host our previous phylogeographic analysis. Our results, based on 31 sequences, showed that the most likely serotype of origin of IBV was Arkansas DPI, which started its spread from Jacksonville city through the state of North Carolina in all directions. This diffusion has shown to occur mostly through long-distance movements. Our results also evidenced that even when we can observe some clusters of similar serotypes in the evolutionary dimension (left panel), it is not possible to observe a clear pattern in the serotype distribution in the geographic space (right panel) (Figure 6).

## Discussion

Several widely distributed IBV serotypes have been identified globally (5, 71). In the United States alone, numerous IBV serotypes have been described after the first report in the early 1930s, such as Georgia, Massachusetts, Arkansas, Connecticut, SE17, and Delaware (47, 70, 72). These studies also demonstrated the existence of a high diversity of IBV strains that were circulating across US farms for more than 60 years (73, 74).

The spike protein, and in particular the S1 subunit, of IBV determines viral tropism. It is the attachment protein that interacts with the receptor molecules on host cells. It is the major viral antigen-stimulating humoral and cell-mediated immunity. It is subject to evolutive changes, and it is highly variable among different serotypes and subtypes. Therefore, most of the genotyping procedures are based on the S1 gene sequencing (71).

Our phylogeographic analysis based on publicly available IBV S1 gene sequences showed that this diversity is mostly concentrated in the eastern part of the United States. Furthermore, this diversity is consistent with the density of poultry farms in the country (75). Likewise, we showed that the most likely ancestral IBV serotype for the studied farms was Arkansas DPI, which has shown to be widely distributed across the study area. This spatial clustering of IBV serotypes evidenced a homogeneous distribution, similar to what was shown by serotype Georgia 13. These broad distributions and continuous presence over time support the hypothesis of Jackwood et al. (76), who proposed that high persistence of Arkansas serotype could be explained by partial protection for that IBV type in the currently used vaccines. This may represent a significant issue since Arkansas type has demonstrated to be quite prone to genetic drift, which could potentially trigger the emergence of unique variants of the IBV virus (77).

Two different primer sets were used for the samples because the S1 gene could not be amplified with the primer set published by Jackwood et al. (47); therefore, a new set of primers was designed. Since two different sets of primers that amplified two non-overlapping segments along the IBV genome were used in this study, the conclusions regarding the phylogenetic analysis should be interpreted considering that the variability provided the different locations of the two sets of amplified segments yield by using two different sets of primers.

One of the main reasons for a pathogen to disseminate through long distances depends directly on its ability to be transported between flocks (78–80). The spread velocity of IBV based on the sequences collected in this study (12.3 km/year) was remarkably lower than that exhibited by other avian diseases, such as highly pathogenic avian influenza virus (HPAIV) subtype H5N1 in Indonesia (325 km/year) (81). These differences in the dissemination of both avian viruses could be related to its mechanisms of transmission, in the case of IBV, mainly mediated by human movement (82, 83), while H5N1 HPAIV avian influenza is mostly triggered by bird migrations (84–87). Previous studies highlighted the importance of airborne spread among nearby farms (88–90); however, more evidence shows that direct contact between birds or with contaminated material and other equipment is common and could indeed be related with between-flock dissemination (91). Further studies are needed to better understand the role of unexplored modes of between-flock transmissions, which also could include indirect contacts among farms, such as the networks formed by food delivery trucks and farm visits, which remains poorly documented (92–94). When we compared sequences from North Carolina to IBV S1 gene sequences collected across the United States for the last 5 years, we observed that 67% of them were genetically related to sequences from other regions such as Alabama, Georgia, and Delaware. The remaining 43% were clustered in three clear groups within North Carolina. Contrasting our results with those of Mondal et al. (70), it is interesting to notice how North Carolina samples from the 1960s are more similar to the sequences found in other states than to the samples sequenced for this study, showing quantitatively IBV genetic variation over time. This variation may be explained by the high accumulation of mutations and recombination events in its viral genome that triggers the emergence of distinct IBV variants (74, 95, 96); however, more studies are needed before better inferences are made.

On the other hand, our phylodynamic model showed no significant covariates for the spread of IBV, which we hypothesized could be directly related to the limited amount of data that was available. This limitation could be surpassed through the collaboration between the poultry industry and researchers to generate more epidemiological and genetic data during future IBV outbreaks.

### Near Real-Time Visualization of Infectious Bronchitis Virus

In recent years, phylodynamics has become a useful approach to study pathogen geographic and evolutionary patterns (97, 98). One of the most recent and impactful examples of how such analysis can be applied to monitor the global SARS-CoV-2 pandemic (REF) that has helped to understand and evaluate the velocity of transmission and diversification of a novel virus worldwide due to the update and release of information in the mentioned near real-time format (99–101). This availability of data has not only helped to study and analyze the diversification ability of the virus in a remarkably short period of time but has also been used to understand its transmission patterns and propose potentially control measures accordingly. In the era of data science, changes in the field of epidemiology have promoted the emergence of a new research area known as outbreak analytics (41), which is rapidly increasing the available scientific knowledge about evolution, geographic spread, and epidemic patterns of several well-known pathogens (42, 102–105). Here, we have shown that the Nextstrain platform could be utilized to monitor the spread of IBV, helping to guide control activities in the field such as informing stakeholders about new viral emergence events and correlate current strain to vaccination strategies.

### Implications for the Poultry Industry

Historically, the most efficient way to prevent and control IBV relied on the use of both inactivated and attenuated live vaccines, which depends on informed decisions regarding the serotypes that are circulating in the field (27). Interestingly, we found that although IBV showed to be suffering an overall gradual decrease in its relative genetic diversity, and despite the known high variability of IBV serotypes found in the field (91), only a few strains are being used as attenuated live vaccines by the poultry industry (106), with Massachusetts (Mass) as the most widely applied (27), which does not provide the necessary cross-protection against IBV. In North Carolina, the currently applied vaccine is MILDVAC-MASS+ARK, which offers coverage against the IBV serotypes Arkansas and Massachusetts (107, 108). However, based on our study, the currently circulating IBV strains in North Carolina are mostly Arkansas DPI, Georgia 13, and Georgia 98, and therefore, the currently applied vaccine is probably not providing sufficient protection against serotypes Georgia 13 and Georgia 98.

Further comparative residue substitution analyses among vaccine and field-originated strains are warranted to better elucidate protection failure or escape mutants. In this study, a number of sequences were collected; however, comprehensive information about the vaccine strains and vaccination programs is needed, and this kind of analyses were not possible to conduct with the information available. Our analyses provide valuable information on the dynamics of IBV field strains and the protection elicited by commercial vaccines. However, vaccination is the only accurate way to determine the degree of protection induced by vaccines is by conducting vaccination and challenge studies in birds. This will give you a better idea on the real protection.

In this study, we provided the stakeholders with an alternative platform via Nextstrain, which provides updated information about IBV serotype circulation and the needed information to ensure targeted vaccination and efficient protection against current IBV serotypes.

## Conclusion

In summary, we have shown an asymmetric pattern in the phylogeographic relationships exhibited by IBV serotypes identified in North Carolina, which imposes a continuous challenge to IBV control. Using the Nextstrain platform, this study provides a near real-time tool for tracking spread in North Carolina, where the collaboration between producers and researchers was the key to gather up-to-date IBV epidemiological information, maintaining the data confidentiality. Thus, accessible visualization tools for the general public represent a powerful tool to ensure adequate stakeholder decision making and government applications to reduce the impact of the disease via targeted control strategies.

## Data Availability Statement

The datasets presented in this study can be found in online repositories. The names of the repository/repositories and accession number(s) can be found in the article/supplementary material.

## Author Contributions

RC and GM conceived the paper ideas. MJ, RC, and GM participated in the design of the study. RC and AB coordinated the IBV sequence data collection. MJ conducted data processing and cleaning. MJ performed the phylogeographic analysis. DR and AC designed and implemented the different security levels that accompanied Nextstrain visualization. MJ, RC, DR, AC, AB, and GM wrote and edited the manuscript. All authors discussed the results and critically reviewed the manuscript.

## Conflict of Interest

The authors declare that the research was conducted in the absence of any commercial or financial relationships that could be construed as a potential conflict of interest.
