# Supplementary material for: Development of a Dissemination Platform for Spatiotemporal and Phylogenetic Analysis of Avian Infectious Bronchitis Virus
Source: Front Vet Sci. 2021 May 4;8:624233. doi: 10.3389/fvets.2021.624233 (PMC8129014; doi:10.3389/fvets.2021.624233)
Supplement: Supplementary file 1 [file Data_Sheet_1.docx]

Supplementary Material

**Supplementary Table 1.** IBV sequences used in this study. Sequences come from the Mississippi Poultry Research and Diagnostic Laboratory (PRDL) and from the North Carolina Veterinary Diagnostic Laboratory System (NCVDLS). In addition, the information about the use of vaccination of each flock was also collected.

| **Laboratory** | **Serotype** | **Vaccine name** | **Collection date** |
| --- | --- | --- | --- |
| PRDL | Arkansas DPI | No data | 10/21/2015 |
| PRDL | Georgia 98 | No data | 11/1/2016 |
| PRDL | Georgia 13 | MILDVAC-MASS+ARK | 3/22/2017 |
| PRDL | Georgia 98 | MILDVAC-MASS+ARK | 4/26/2017 |
| PRDL | Georgia 98 | MILDVAC-MASS+ARK | 4/26/2017 |
| PRDL | Georgia 98 | MILDVAC-MASS+ARK | 4/26/2017 |
| PRDL | Georgia 98 | MILDVAC-MASS+ARK | 4/26/2017 |
| PRDL | Georgia 98 | MILDVAC-MASS+ARK | 4/26/2017 |
| PRDL | Arkansas DPI | MILDVAC-MASS+ARK | 4/26/2017 |
| PRDL | Georgia 98 | MILDVAC-MASS+ARK | 4/26/2017 |
| PRDL | Georgia 13 | MILDVAC-MASS+ARK | 5/4/2017 |
| PRDL | Georgia 13 | No data | 5/4/2017 |
| PRDL | Georgia 13 | MILDVAC-MASS+ARK | 6/20/2017 |
| PRDL | Arkansas DPI | MILDVAC-MASS+ARK | 6/20/2017 |
| PRDL | Georgia 98 | MILDVAC-MASS+ARK | 6/20/2017 |
| PRDL | Georgia 98 | MILDVAC-MASS+ARK | 6/20/2017 |
| PRDL | Arkansas DPI | MILDVAC-MASS+ARK | 6/20/2017 |
| PRDL | Arkansas DPI | MILDVAC-MASS+ARK | 6/20/2017 |
| PRDL | Arkansas DPI | No data | 7/5/2017 |
| PRDL | Arkansas DPI | No data | 7/5/2017 |
| PRDL | Arkansas DPI | No data | 7/5/2017 |
| PRDL | Georgia 98 | No data | 7/5/2017 |
| PRDL | Georgia 98 | No data | 7/5/2017 |
| PRDL | Georgia 13 | MILDVAC-MASS+ARK | 7/20/2017 |
| PRDL | Georgia 13 | MILDVAC-MASS+ARK | 7/20/2017 |
| PRDL | Georgia 13 | MILDVAC-MASS+ARK | 7/20/2017 |
| PRDL | Georgia 13 | MILDVAC-MASS+ARK | 4/4/2018 |
| PRDL | Georgia 13 | MILDVAC-MASS+ARK | 4/4/2018 |
| PRDL | Georgia 13 | MILDVAC-MASS+ARK | 4/4/2018 |
| PRDL | Georgia 13 | MILDVAC-MASS+ARK | 4/4/2018 |
| PRDL | Arkansas DPI | MILDVAC-MASS+ARK | 4/18/2018 |
| PRDL | Arkansas DPI | MILDVAC-MASS+ARK | 4/18/2018 |
| PRDL | Arkansas DPI | MILDVAC-MASS+ARK | 4/18/2018 |
| PRDL | Arkansas DPI | MILDVAC-MASS+ARK | 4/18/2018 |
| PRDL | Georgia 13 | MILDVAC-MASS+ARK | 5/2/2018 |
| PRDL | Georgia 13 | MILDVAC-MASS+ARK | 5/2/2018 |
| PRDL | Arkansas DPI | MILDVAC-MASS+ARK | 6/5/2018 |
| PRDL | Arkansas DPI | MILDVAC-MASS+ARK | 6/5/2018 |
| PRDL | Arkansas DPI | MILDVAC-MASS+ARK | 11/29/2018 |
| PRDL | Arkansas DPI | MILDVAC-MASS+ARK | 11/29/2018 |
| PRDL | Georgia 13 | MILDVAC-MASS+ARK | 11/29/2018 |
| PRDL | Georgia 13 | MILDVAC-MASS+ARK | 11/29/2018 |
| PRDL | No data | MILDVAC-MASS+ARK | 11/29/2018 |
| PRDL | Georgia 13 | MILDVAC-MASS+ARK | 11/29/2018 |
| PRDL | Arkansas DPI | MILDVAC-MASS+ARK | 11/29/2018 |
| PRDL | Arkansas DPI | MILDVAC-MASS+ARK | 11/29/2018 |
| PRDL | Georgia 13 | MILDVAC-MASS+ARK | 11/29/2018 |
| PRDL | Georgia 13 | MILDVAC-MASS+ARK | 12/11/2018 |
| PRDL | Arkansas DPI | MILDVAC-MASS+ARK | 12/14/2018 |
| PRDL | Georgia 13 | MILDVAC-MASS+ARK | 12/29/2018 |
| PRDL | Arkansas DPI | MILDVAC-MASS+ARK | 1/3/2019 |
| PRDL | Georgia 13 | MILDVAC-MASS+ARK | 1/3/2019 |
| PRDL | Georgia 13 | No data | 2/21/2019 |
| PRDL | Georgia 13 | No data | 2/21/2019 |
| PRDL | Georgia 13 | No data | 3/12/2019 |
| PRDL | Georgia 13 | No data | 3/13/2019 |
| PRDL | Georgia 13 | No data | 3/13/2019 |
| PRDL | Georgia 13 | No data | 3/13/2019 |
| PRDL | Georgia 13 | No data | 3/13/2019 |
| PRDL | Georgia 13 | No data | 3/13/2019 |
| PRDL | Georgia 13 | No data | 3/13/2019 |
| PRDL | Georgia 13 | No data | 3/25/2019 |
| PRDL | Georgia 13 | No data | 3/25/2019 |
| PRDL | Georgia 13 | No data | 4/5/2019 |
| PRDL | Georgia 13 | No data | 4/5/2019 |
| PRDL | Georgia 13 | No data | 4/5/2019 |
| PRDL | Arkansas DPI | No data | 4/5/2019 |
| PRDL | DMV/1639 | No data | 4/5/2019 |
| PRDL | Georgia 13 | No data | 4/10/2019 |
| PRDL | Arkansas DPI | No data | 4/10/2019 |
| PRDL | DMV/1639 | No data | 4/10/2019 |
| PRDL | DMV/1639 | No data | 4/16/2019 |
| PRDL | Arkansas DPI | No data | 4/16/2019 |
| PRDL | Arkansas DPI | No data | 4/16/2019 |
| PRDL | Arkansas DPI | No data | 4/16/2019 |
| PRDL | Arkansas DPI | No data | 4/16/2019 |
| PRDL | Georgia 13 | No data | 4/17/2019 |
| PRDL | Arkansas DPI | No data | 4/17/2019 |
| PRDL | Arkansas DPI | No data | 4/25/2019 |
| NCVDLS | Arkansas_DPI | No data | 4/25/2019 |
| NCVDLS | Arkansas_DPI | No data | 4/25/2019 |
| NCVDLS | Arkansas_DPI | No data | 4/25/2019 |
| NCVDLS | Arkansas_DPI | No data | 4/25/2019 |
| NCVDLS | Arkansas_DPI | No data | 4/25/2019 |
| NCVDLS | Arkansas_DPI | No data | 4/25/2019 |
| NCVDLS | Arkansas_DPI | No data | 4/25/2019 |
| NCVDLS | Arkansas_DPI | No data | 4/25/2019 |
| NCVDLS | Arkansas_DPI | No data | 4/25/2019 |
| NCVDLS | Arkansas_DPI | No data | 4/25/2019 |
| NCVDLS | Arkansas_DPI | No data | 4/25/2019 |
| NCVDLS | Arkansas_DPI | No data | 4/25/2019 |
| NCVDLS | Arkansas_DPI | No data | 4/25/2019 |
| NCVDLS | Arkansas_DPI | No data | 4/25/2019 |
| NCVDLS | Arkansas_DPI | No data | 4/25/2019 |
| NCVDLS | Arkansas_DPI | No data | 4/25/2019 |
| NCVDLS | Arkansas_DPI | No data | 4/25/2019 |
| NCVDLS | Arkansas_DPI | No data | 4/25/2019 |
| NCVDLS | Arkansas_DPI | No data | 4/25/2019 |
| NCVDLS | Arkansas_DPI | No data | 4/25/2019 |
| NCVDLS | Arkansas_DPI | No data | 4/25/2019 |
| NCVDLS | Arkansas_DPI | No data | 4/25/2019 |
| NCVDLS | Arkansas_DPI | No data | 4/25/2019 |
| NCVDLS | Arkansas_DPI | No data | 4/25/2019 |
| NCVDLS | Arkansas_DPI | No data | 4/25/2019 |
| NCVDLS | Arkansas_DPI | No data | 4/25/2019 |
| NCVDLS | Arkansas_DPI | No data | 4/25/2019 |
| NCVDLS | Arkansas_DPI | No data | 4/25/2019 |
| NCVDLS | Arkansas_DPI | No data | 4/25/2019 |
| NCVDLS | Arkansas_DPI | No data | 4/25/2019 |
| NCVDLS | Arkansas_DPI | No data | 4/25/2019 |
| NCVDLS | Arkansas_DPI | No data | 4/25/2019 |
| NCVDLS | Arkansas_DPI | No data | 4/25/2019 |
| NCVDLS | Arkansas_DPI | No data | 4/25/2019 |
| NCVDLS | Arkansas_DPI | No data | 4/25/2019 |
| NCVDLS | Arkansas_DPI | No data | 4/25/2019 |
| NCVDLS | Arkansas_DPI | No data | 4/25/2019 |
| NCVDLS | Arkansas_DPI | No data | 4/25/2019 |
| NCVDLS | Arkansas_DPI | No data | 4/25/2019 |
| NCVDLS | Arkansas_DPI | No data | 4/25/2019 |
| NCVDLS | Arkansas_DPI | No data | 4/25/2019 |
| NCVDLS | Arkansas_DPI | No data | 4/25/2019 |
| NCVDLS | Arkansas_DPI | No data | 4/25/2019 |
| NCVDLS | Arkansas_DPI | No data | 4/25/2019 |
| NCVDLS | Arkansas_DPI | No data | 4/25/2019 |
| NCVDLS | Arkansas_DPI | No data | 4/25/2019 |
| NCVDLS | Arkansas_DPI | No data | 4/25/2019 |
| NCVDLS | Arkansas_DPI | No data | 4/25/2019 |
| NCVDLS | Arkansas_DPI | No data | 4/25/2019 |
| NCVDLS | Arkansas_DPI | No data | 4/25/2019 |
| NCVDLS | Arkansas_DPI | No data | 4/25/2019 |
| NCVDLS | Arkansas_DPI | No data | 4/25/2019 |
| NCVDLS | Arkansas_DPI | No data | 4/25/2019 |

**Supplementary Table 2.** Genbank IBV S1 gene sequences collected and posted from farms located in the United States.

| **GenBank ID** | **Serotype** | **State** |
| --- | --- | --- |
| AF006624 | Arkansas DPI | Delaware |
| AF006625 | Arkansas DPI | Delaware |
| AF169856 | Arkansas DPI | Delaware |
| AF169858 | Arkansas DPI | Delaware |
| AF169859 | Arkansas DPI | Delaware |
| AF169860 | Arkansas DPI | Delaware |
| AF274437 | Georgia 98 | Georgia |
| AF510564 | AL 11271 97 | Alabama |
| AF520605 | WI 5340 98 | Alabama |
| AF520607 | IA 10624 99 | Alabama |
| AY027541 | CU83074 | New York |
| AY101766 | AL 9021 97 | Alabama |
| AY789945 | PA 5083 99 | Delaware |
| AY942737 | Cal99 | California |
| EU283049 | Arkansas DPI | Georgia |
| GU437857 | GPL8121 | Georgia |
| GU437858 | GPL8225 | Georgia |
| GU437864 | GPL8264 | Georgia |
| JF774063 | IBV CAL99 | California |
| JQ964060 | Massachusetts | Mississippi |
| JQ964061 | SE17 | Arkansas DPI |
| JQ964062 | Massachusetts | Arkansas DPI |
| JQ964063 | Massachusetts | Alabama |
| JQ964064 | Connecticut | Mississippi |
| JQ964065 | Massachusetts | Alabama |
| JQ964066 | SE17 | Indiana |
| JQ964067 | SE17 | North Carolina |
| JQ964068 | Massachusetts | Mississippi |
| JQ964069 | Massachusetts | Alabama |
| JQ964070 | SE17 | Tennessee |
| JQ964071 | SE17 | Mississippi |
| JQ964072 | Massachusetts | Hawaii |
| KX529778 | Arkansas DPI | Georgia |
| L14069 | Gray | Delaware |
| L14070 | JMK | Delaware |
| U77298 | DE072 | Delaware |

**Supplementary Table 3.** Model selection results based on the best-fit molecular clock model. The model that appears in bold represents the best model chosen according to the Bayesian Information Criterion (BIC). AIC= Akaike Information Criterion, AICc= Corrected Akaike Information Criterion.

| **Model** | **AIC** | **AICc** | **BIC** |
| --- | --- | --- | --- |
| F81+F | 60434.5 | 60526.7 | 61810 |
| F81+F+G4 | 57939.5 | 58032.4 | 59320.4 |
| F81+F+I | 60403.3 | 60496.2 | 61784.1 |
| F81+F+I+G4 | 57941.5 | 58035.2 | 59327.7 |
| GTR+F | 60089.4 | 60185.6 | 61492 |
| GTR+F+G4 | 57569.6 | 57666.5 | 58977.5 |
| GTR+F+I | 60057.8 | 60154.7 | 61465.7 |
| GTR+F+I+G4 | 57571.4 | 57669.2 | 58984.8 |
| HKY+F | 60105.1 | 60198 | 61486 |
| HKY+F+G4 | 57586.5 | 57680.3 | 58952.8 |
| HKY+F+I | 60072.8 | 60166.5 | 61459.1 |
| HKY+F+I+G4 | 57588.4 | 57683 | 58980.1 |
| JC | 60712.9 | 60802.7 | 62072.1 |
| JC+G4 | 58314.3 | 58404.9 | 59679 |
| JC+I | 60684.1 | 60774.6 | 62048.7 |
| JC+I+G4 | 58316.5 | 58407.9 | 59686.6 |
| K2P | 60512.7 | 60603.3 | 61877.3 |
| K2P+G4 | 58084.2 | 58175.5 | 59454.2 |
| K2P+I | 60483.6 | 60574.9 | 61853.6 |
| K2P+I+G4 | 58086.1 | 58178.2 | 59461.5 |
| K3P | 60512.4 | 60603.7 | 61882.4 |
| K3P+G4 | 58085.1 | 58177.2 | 59460.5 |
| K3P+I | 60483.3 | 60575.4 | 61858.7 |
| K3P+I+G4 | 58086.9 | 58179.8 | 59467.8 |
| K3Pu+F | 60088.1 | 60181.8 | 61474.3 |
| K3Pu+F+G4 | 57568.5 | 57663.1 | 58960.2 |
| K3Pu+F+I | 60055.8 | 60150.3 | 61447.5 |
| K3Pu+F+I+G4 | 57570.4 | 57665.7 | 58967.5 |
| SYM | 60422.5 | 60516.2 | 61808.8 |
| SYM+G4 | 57932.1 | 58026.6 | 59323.8 |
| SYM+I | 60393.3 | 60487.8 | 61785 |
| SYM+I+G4 | 57934 | 58029.3 | 59331.1 |
| TIM+F | 60088.5 | 60183.1 | 61480.2 |
| TIM+F+G4 | 57568.3 | 57663.6 | 58965.4 |
| TIM+F+I | 60056.6 | 60151.9 | 61453.7 |
| TIM+F+I+G4 | 57570.2 | 57666.3 | 58972.7 |
| TIM2+F | 60097.5 | 60192 | 61489.2 |
| TIM2+F+G4 | 57579.1 | 57674.4 | 58976.2 |
| TIM2+F+I | 60065.9 | 60161.2 | 61463 |
| TIM2+F+I+G4 | 57580.9 | 57677 | 58983.4 |
| TIM2e | 60498.4 | 60590.5 | 61873.8 |
| TIM2e+G4 | 58054.6 | 58147.5 | 59435.4 |
| TIM2e+I | 60468.6 | 60561.5 | 61849.4 |
| TIM2e+I+G4 | 58056.4 | 58150.1 | 59442.7 |
| TIM3+F | 60107.4 | 60201.9 | 61499.1 |
| TIM3+F+G4 | 57588.4 | 57683.8 | 58985.6 |
| TIM3+F+I | 60075.3 | 60170.6 | 61472.4 |
| TIM3+F+I+G4 | 57590.3 | 57686.4 | 58992.8 |
| TIM3e | 60441.7 | 60533.8 | 61817.1 |
| TIM3e+G4 | 57971.1 | 58064 | 59352 |
| TIM3e+I | 60413.3 | 60506.3 | 61794.2 |
| TIM3e+I+G4 | 57973 | 58066.7 | 59359.3 |
| TIMe | 60514 | 60606.1 | 61889.4 |
| TIMe+G4 | 58086.9 | 58179.8 | 59467.7 |
| TIMe+I | 60484.9 | 60577.9 | 61865.8 |
| TIMe+I+G4 | 58088.7 | 58182.4 | 59475 |
| TN+F | 60105.6 | 60199.4 | 61491.9 |
| TN+F+G4 | 57586.5 | 57681 | 58978.2 |
| TN+F+I | 60073.6 | 60168.2 | 61465.3 |
| TN+F+I+G4 | 57588.4 | 57683.7 | 58985.5 |
| TNe | 60514.3 | 60605.6 | 61884.3 |
| TNe+G4 | 58086 | 58178.1 | 59461.4 |
| TNe+I | 60485.2 | 60577.4 | 61860.7 |
| TNe+I+G4 | 58087.8 | 58180.7 | 59468.7 |
| TPM2+F | 60096.9 | 60190.7 | 61483.2 |
| TPM2+F+G4 | 57579.6 | 57674.1 | 58971.3 |
| TPM2+F+I | 60065.1 | 60159.6 | 61456.8 |
| TPM2+F+I+G4 | 57581.4 | 57676.7 | 58978.5 |
| TPM2u+F | 60096.9 | 60190.7 | 61483.2 |
| TPM2u+F+G4 | 57579.6 | 57674.1 | 58971.3 |
| TPM2u+F+I | 60065.1 | 60159.6 | 61456.8 |
| TPM2u+F+I+G4 | 57581.4 | 57676.7 | 58978.5 |
| TPM3+F | 60106.9 | 60200.6 | 61493.2 |
| TPM3+F+G4 | 57588.5 | 57683 | 58980.2 |
| TPM3+F+I | 60074.5 | 60169 | 61466.2 |
| TPM3+F+I+G4 | 57590.3 | 57685.6 | 58987.4 |
| TPM3u+F | 60106.9 | 60200.6 | 61493.2 |
| TPM3u+F+G4 | 57588.5 | 57683 | 58980.2 |
| TPM3u+F+I | 60074.5 | 60169 | 61466.2 |
| TPM3u+F+I+G4 | 57590.3 | 57685.6 | 58987.4 |
| TVM+F | 60088.9 | 60184.2 | 61486 |
| TVM+F+G4 | 57570 | 57666.2 | 58972.6 |
| TVM+F+I | 60057 | 60153.2 | 61459.6 |
| TVM+F+I+G4 | 57571.8 | 57668.8 | 58979.8 |
| TVMe | 60420.7 | 60513.7 | 61801.6 |
| TVMe+G4 | 57930.1 | 58023.8 | 59316.4 |
| TVMe+I | 60391.5 | 60485.2 | 61777.8 |
| TVMe+I+G4 | 57932 | 58026.5 | 59323.7 |

**Supplementary Table 4.** Description and sources of the predictive factors used in this study.

| **Predictors** | **Unit** | **Source** |
| --- | --- | --- |
| Temperature | °C | <https://neo.sci.gsfc.nasa.gov/> |
| Precipitation | mm | <https://climate.northwestknowledge.net/TERRACLIMATE/index_directDownloads.php> |
| Elevation | m | <https://www.usgs.gov/> |
| Vegetation (EVI) | EVI index | <https://modis.gsfc.nasa.gov/> |
| Soil humidity | mm | [http://worldgrids.org](http://worldgrids.org/) |
| Runoff | mm | <https://climate.northwestknowledge.net/TERRACLIMATE/index_directDownloads.php> |
| Chicken density | Ind/km^2^ | <https://dataverse.harvard.edu/dataverse/glw_3> |
| Distance to road | m | <https://catalog.data.gov/> |

**Supplementary Table 5.** IBV sequences used in Nextstrain visualization.

| **Serotype** | **Vaccine applied** | **Collection date** |
| --- | --- | --- |
| Georgia_13 | MILDVAC-MASS+ARK | 1/3/2019 |
| Arkansas_DPI | MILDVAC-MASS+ARK | 12/14/2018 |
| Georgia_13 | MILDVAC-MASS+ARK | 12/11/2018 |
| Georgia_13 | MILDVAC-MASS+ARK | 12/11/2018 |
| Arkansas_DPI | MILDVAC-MASS+ARK | 11/29/2018 |
| Arkansas_DPI | MILDVAC-MASS+ARK | 11/29/2018 |
| Georgia_13 | MILDVAC-MASS+ARK | 11/29/2018 |
| Georgia_13 | MILDVAC-MASS+ARK | 11/29/2018 |
| Georgia_13 | MILDVAC-MASS+ARK | 11/29/2018 |
| Arkansas_DPI | MILDVAC-MASS+ARK | 6/5/2018 |
| Arkansas_DPI | MILDVAC-MASS+ARK | 6/5/2018 |
| Georgia_13 | MILDVAC-MASS+ARK | 5/2/2018 |
| Georgia_13 | MILDVAC-MASS+ARK | 5/2/2018 |
| Arkansas_DPI | MILDVAC-MASS+ARK | 4/18/2018 |
| Arkansas_DPI | MILDVAC-MASS+ARK | 4/18/2018 |
| Arkansas_DPI | MILDVAC-MASS+ARK | 4/18/2018 |
| Arkansas_DPI | MILDVAC-MASS+ARK | 4/18/2018 |
| Georgia_13 | MILDVAC-MASS+ARK | 4/4/2018 |
| Georgia_13 | MILDVAC-MASS+ARK | 4/4/2018 |
| Georgia_13 | MILDVAC-MASS+ARK | 7/20/2017 |
| Georgia_13 | MILDVAC-MASS+ARK | 7/20/2017 |
| Georgia_13 | MILDVAC-MASS+ARK | 7/20/2017 |
| Georgia_13 | MILDVAC-MASS+ARK | 6/20/2017 |
| Georgia_13 | MILDVAC-MASS+ARK | 5/4/2017 |
| Georgia_98 | MILDVAC-MASS+ARK | 4/26/2017 |
| Georgia_98 | MILDVAC-MASS+ARK | 4/26/2017 |
| Georgia_98 | MILDVAC-MASS+ARK | 4/26/2017 |
| Georgia_98 | MILDVAC-MASS+ARK | 4/26/2017 |
| Georgia_98 | MILDVAC-MASS+ARK | 4/26/2017 |
| Arkansas_DPI | MILDVAC-MASS+ARK | 4/26/2017 |
| Georgia_13 | MILDVAC-MASS+ARK | 3/22/2017 |
